# Concept of a fully-implantable system to monitor tumor recurrence

**DOI:** 10.1038/s41598-023-43226-3

**Published:** 2023-09-29

**Authors:** Anna Schaufler, Ahmed Y. Sanin, I. Erol Sandalcioglu, Karl Hartmann, Roland S. Croner, Aristotelis Perrakis, Thomas Wartmann, Axel Boese, Ulf D. Kahlert, Igor Fischer

**Affiliations:** 1 Molecular and Experimental Surgery, Clinic for General-, Visceral-, Vascular - and Transplant Surgery, Faculty of Medicine, Otto-von-Guericke University Magdeburg, 39120 Magdeburg, Germany; 2 Department of Neurosurgery, Otto-von-Guericke University Magdeburg, 39120 Magdeburg, Germany; 3 INKA Health Tech Innovation Lab., Medical Faculty, Otto-von-Guericke University Magdeburg, 39120 Magdeburg, Germany; 4 Research Campus STIMULATE, Otto-von-Guericke University Magdeburg, 39120 Magdeburg, Germany; 5 Department of Neurosurgery, Medical Faculty and University Hospital Düsseldorf, Heinrich-Heine-University Düsseldorf, 40225 Düsseldorf, Germany

**Keywords:** Surgical oncology, Biomedical engineering

## Abstract

Current treatment for glioblastoma includes tumor resection followed by radiation, chemotherapy, and periodic post-operative examinations. Despite combination therapies, patients face a poor prognosis and eventual recurrence, which often occurs at the resection site. With standard MRI imaging surveillance, histologic changes may be overlooked or misinterpreted, leading to erroneous conclusions about the course of adjuvant therapy and subsequent interventions. To address these challenges, we propose an implantable system for accurate continuous recurrence monitoring that employs optical sensing of fluorescently labeled cancer cells and is implanted in the resection cavity during the final stage of tumor resection. We demonstrate the feasibility of the sensing principle using miniaturized system components, optical tissue phantoms, and porcine brain tissue in a series of experimental trials. Subsequently, the system electronics are extended to include circuitry for wireless energy transfer and power management and verified through electromagnetic field, circuit simulations and test of an evaluation board. Finally, a holistic conceptual system design is presented and visualized. This novel approach to monitor glioblastoma patients is intended to early detect recurrent cancerous tissue and enable personalization and optimization of therapy thus potentially improving overall prognosis.

## Introduction

The propensity of malignant tissue to accumulate substances with fluorescent properties has been known for many decades. Fluorescein has been reported as early as 1947^1,2^, and the same tendency was observed on porphyrins in 1948^3,4^. Many other markers have been discovered since and have entered diagnostic and clinical use^5–8^ etc. 5-aminolevulinic acid (5-ALA)-induced porphyrins have been in use as a marker in fluorescence-guided surgery of primary brain tumors since at least 1998^9^.

Malignant gliomas are a major primary brain tumor type. Despite years of research, their etiology and origins are still unknown^10^. Glioblastoma multiforme (GBM) is their most malignant variant, accounting for almost 50% of malignant glioma, with an incidence of about 3.2 per year and 100,000 population in western countries^11^. Glioblastoma show a highly infiltrating growth pattern, rendering complete resection virtually impossible. Therefore, current treatment protocols combine resection with radiotherapy and chemotherapy. Still, the prognosis is poor: For the most common genetic form, IDH-wildtype, the median survival is only 10-15 months after surgery, and even for the less aggressive mutant form, which accounts for about 10% of the GBM cases, it is only 24–31 months^12,13^. In spite of maximal primary resection and combination therapy, the 5-year survival rate for recurrent glioblastoma remains below 5%, with tumor recurrence being the leading cause of mortality occurring at a recurrence rate of approximately 90%. Here, approximately 70% of patients experience recurrence within the first year after diagnosis and in 90% of cases, disease progression manifests locally at the primary site within a boundary of less than 2 cm ^14–16^.

As GBM are bound to reappear, despite therapy, patients are required to undergo frequent MRI examinations, typically every three months, to decide on the further course of treatment. However, there are known difficulties in neuro-radiological assessment using conventional MRI. Therapy-induced tissue changes such as radiation necrosis, edema, or effects like reduced contrast uptake caused by anti-angiogenic drugs often manifest as pseudoprogression and pseudoresponse in the images^17^. To address this problem, various advanced MRI imaging modalities for the surveillance of GBM patients are being investigated and described. These modalities include MR perfusion, diffusion-weighted imaging (DWI), chemical exchange saturation transfer, and nuclear medicine imaging, in addition to traditional MRI^18^. Some of these methods achieve excellent sensitivity and specificity in tissue differentiation, for example, up to 100% in DWI. By eliminating the need for a second reference measurement after 4–8 weeks, these techniques indirectly expedite the detection of recurrence, allowing for faster diagnosis ^17^. A novel MRI technique, so called Vascular Architecture Mapping (VAM) was introduced by Stadlbauer et al.^19^ in 2019. This technique utilizes spin-echo MRI to visualize small vessels, which are particularly involved in neovascularization. When combined with maps of macrovascular perfusion obtained from perfusion MRI, imaging biomarkers for glioblastomas with small volumes could be extracted. On average, tumors were detected 147 days earlier with the new method compared to conventional MRI imaging. Nevertheless, for the purpose of immediate detection of tumor recurrence, patients would need continuous monitoring. In practice, this is not feasible with conventional imaging. Neither could patients spend the rest of their lives in the MRI scanner, nor could our health system afford an MRI scanner per patient.

Another approach to diagnosis could be liquid biopsies, which involve examining body fluids such as blood for the presence of circulating glioblastoma biomarkers, such as tumor cells, cell-free nucleic acids, or proteins. This approach could overcome the limitations of imaging diagnoses by enabling serial sampling. Although GBM-specific biomarkers have been found in patients, there is a lack of technologies that can perform the extraction of these markers consistently and reliably. Furthermore, the blood-brain barrier poses a significant challenge, as it prevents the relevant molecules from crossing from the brain into the blood ^20^.

Moreover, approaches are being developed to predict early recurrence and utilize this information to create personalized treatment plans. Wang et al.^21^ implemented a predictive model that extracts radiomics features from preoperative MRI (with an area under the curve of 0.84 in the training cohort). Jin et al.^22^ found that features from diffusion-weighted MRI imaging, particularly fractional anisotropy, serve as a predictor for the presence of subclinical recurrence. A review by Corr et al.^23^ evaluated studies that used radiogenomics as a basis for predicting early recurrence, identifying indicators for tumor subtype, chemotherapy responsiveness, overall survival, and progression-free survival.

Nevertheless, a higher frequency of monitoring sessions could lead to earlier detection of tumor recurrence, more timely intervention, and improvement in patients’ life quality and life expectancy. For that purpose, we envision an implantable medical device for early detection of glioblastoma recurrence. The device relies on the above-mentioned protoporphyrin IX (PPIX) fluorescence and would be implanted at the end of surgery inside the cavity left after the tumor is removed.

The idea of having a device in patients’ body, which could give visual feedback regarding pathological developments, is not new. For the digestive tract, an ingestible video camera in form of a pill has been around for two decades^24^. However, since it passes the tract within hours, its power supply is not designed for long-term monitoring. In recent years, there have been some advances in implantable sensors for cancer and/or brain monitoring^25–27^. For the purpose of detecting glioma recurrence, high-resolution imaging or video, requiring complex miniaturized hardware and with high power consumption might be not necessary. Simple detection of fluorescence level, or even a binary detection, is enough to provide early warning to the patient and prompt them to consult a specialist for a thorough examination, including MRI. Reduction in complexity and power requirements allows for further miniaturization of the implant and better electro magnetic-compatibility with MRI machines.

The novel concept for high-frequent, high-accuracy monitoring of recurrent cancer tissue after glioblastoma resection described in this paper centers on an implant to detect fluorescently labeled cancer cells. This paper aims to demonstrate the validity of the proposed measuring principle and conceptualize the initial design of a feasible system implementation. This concept is intended to enable the patient to monitor glioblastoma recurrence autonomously at home or in a local healthcare facility with superior monitoring frequency and accuracy. Besides the aspect of a timely re-resection, the gain of information necessary for the control and adjustment of adjuvant therapy, which can mean a significant improvement of the prognosis by an individual adjustment, should be emphasized.

### Conceptual design

**Figure 1:**
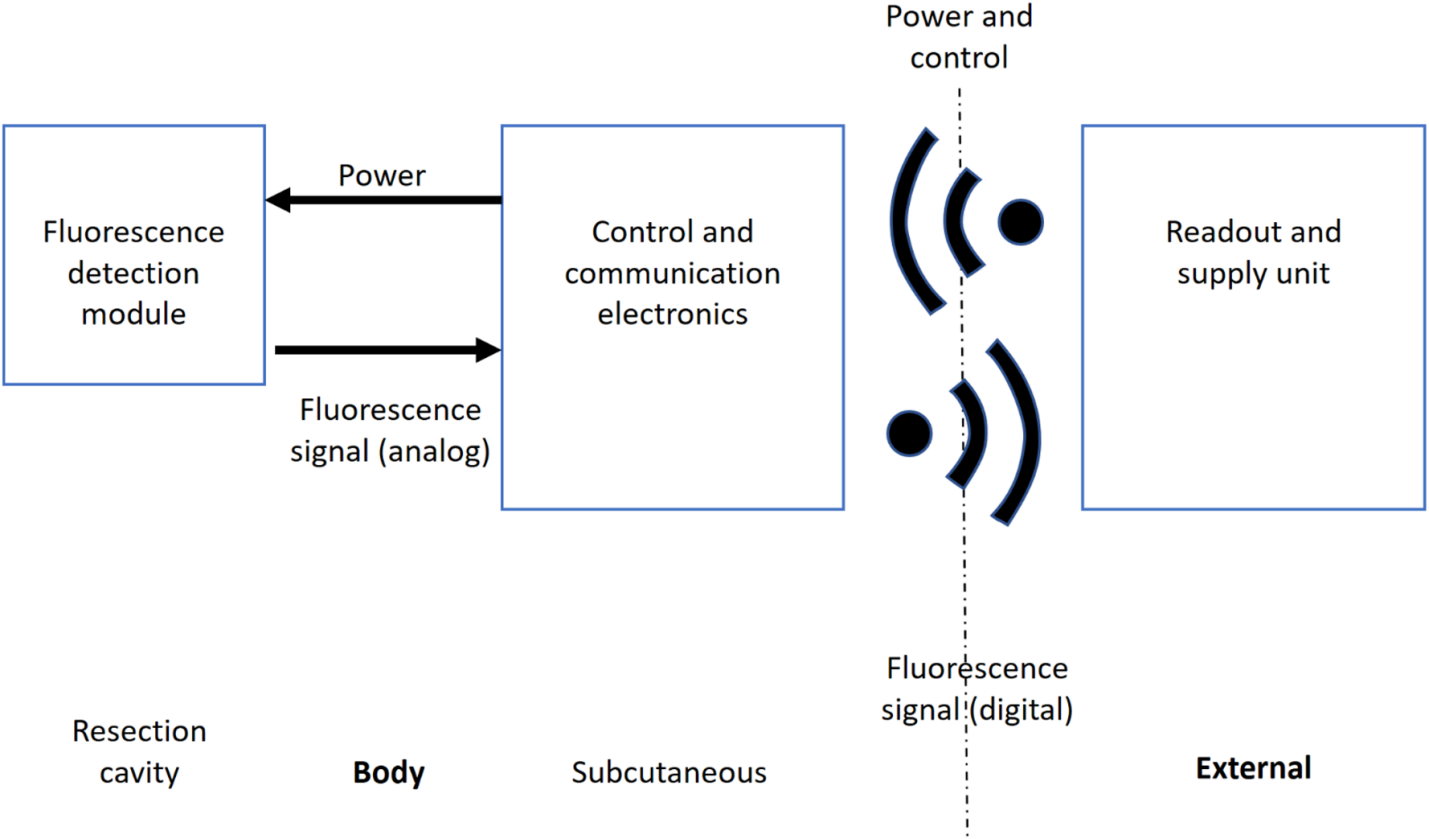
High-level diagram of the components and their interaction.

We propose a bi-modular design for our measurement system, inspired by modern Cochlear and Deep Brain Stimulation implants. In those designs, the sensitive system component is placed directly at the interaction site with the human anatomy, while additional electronics required for its function are placed at a more anatomically accessible and lower-risk location. The proposed system is intended to be introduced in the final phase of the initial tumor removal procedure, comprised of two modules connected by a thin, flexible cable. The fluorescence detection module, which contains fluorescence excitation light sources and selective light detection sensors, is to be placed directly in the resection cavity. The control and communication electronics are located off-site from the resection cavity and placed in a separate module below the scalp on the skull bone. This computing module is responsible for managing the measurements, as well as transcutaneous energy delivery and communication to and from an extracorporeal readout and supply unit. The interaction between the computing module and the extracorporeal readout and supply unit is wireless, while the communication between the computing module and the fluorescence detection module occurs through the above mentioned thin flexible cable. A high-level diagram showing the components and their interaction is shown in Fig. 1.

## Materials and methods

5-ALA, which is an essential complementary element of the monitoring approach, is metabolized in glioblastoma cells to the fluorescent compound protoporphyrin IX (PPIX). PPIX absorbs blue light of a wavelength of 405 nm and emits a light signal at 635 nm central wavelength. Conventional fluorescence-based imaging utilizes powerful light sources and complex optical systems with sophisticated, extensive filter arrangements in order to provoke the highest possible quantum yield in the fluorescent agent molecules and selectively capture relevant emitted light components. For the optical detector presented here, high requirements on the reduction of size and power consumption while maintaining sufficient sensitivity have to be met in the choice of the employed components. Due to high efficiency (lumen/watts) and availability in various dimensional designs, light emitting diodes (LEDs) were therefore specified as light sources and photodiodes with enlarged photosensitive active area as sensory elements in the detector. Three experimental investigation setups were conducted consecutively to address feasibility aspects in this context:

1. Can physiologically relevant concentrations of PPIX in bio-tissue be excited by a restricted number of small-sized power-limited LEDs to the point where the emitted fluorescent light can be recorded by an optical filter-covered photodiode?
2. Can this light source-sensor combination excite and detect fluorescence in samples with realistic optical properties of tissue at a comparable resolution to conventional monitoring methods?
3. Can a signal be detected in biological tissue, considering autofluorescence phenomena and morphological heterogeneities in the background?

The implant concept was then translated into a conceptual design incorporating all electronic components necessary for its function. This step included the extension of the measurement electronics tested in the preceding experiments by a power management circuit for wireless charging, based on field simulations of electromagnetic energy transmission, circuit simulations of the charging circuit, and test of a charging evaluation board. All electronic system elements were finally combined on a board layout and visualized in a 3D modulation of the board with housing.

### Measurement electronics configuration

**Figure 2:**
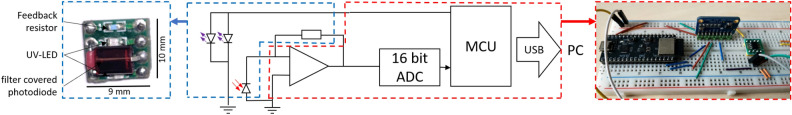
Schematic representation of the electronic measurement circuit.

Two LEDs (SM1206UV-405-IL, Bivar Inc., Irvine, California, USA) with a central wavelength of 405 nm were soldered above and below a photodiode (BPW 34 S, OSRAM Opto Semiconductors GmbH, Regensburg, Germany) with 7.02 mm$^{2}$ radiant area and 60$^{\circ }$ half angle on a universal adapter for SMD components (Fig. 2, left-hand side). The sensitive sensor area was covered with two layers of a red lightning gel filter. The photodiode was connected to a transimpedance amplifier in photovoltaic mode where a 3 megaohm feedback resistor, also located on the SMD adapter, was employed for signal amplification. The measurement electronics further included a 16 Bit analog to digital converter (ADC) and a microcontroller unit (MCU) for measurement execution and recording (Fig. 2). Measurement data was transferred to a personal computer via a serial interface where they were stored for further analysis using MATLAB (MATLAB Release2022b, The MathWorks, Inc., Natick, Massachusetts, USA).

### Measurement setup - liquid samples

**Figure 3:**
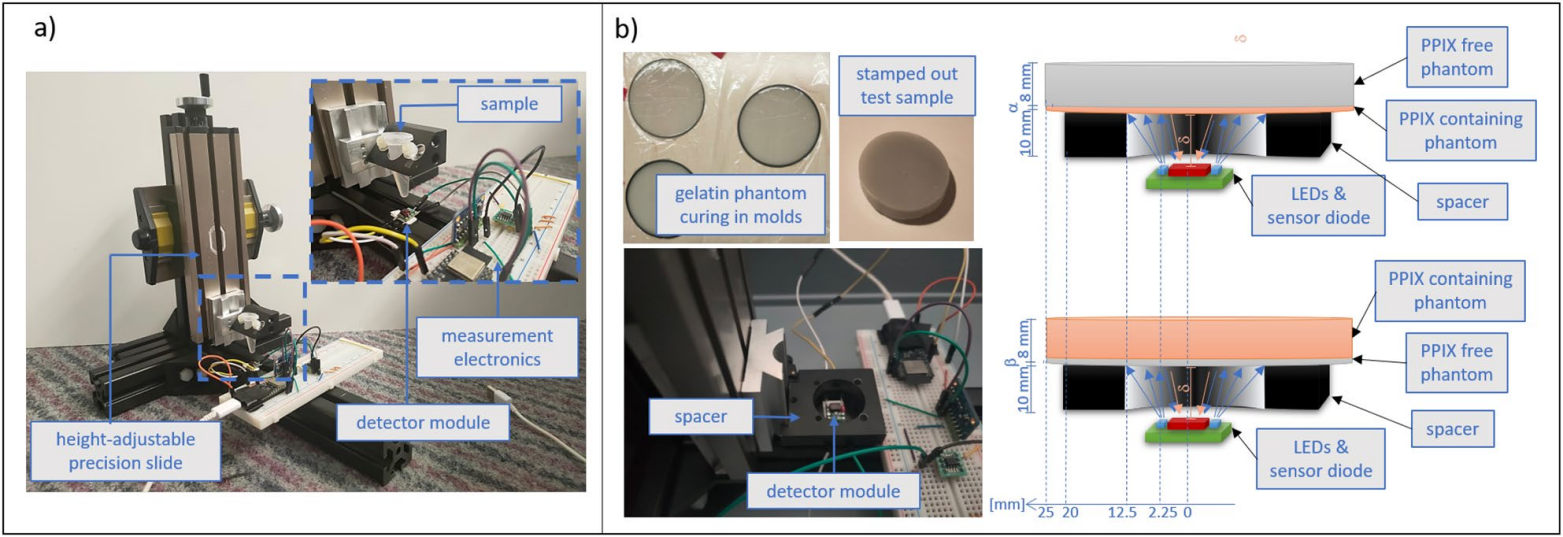
Experimental setup: (**a**) setup with aligned height adjustable holder for liquid samples; (**b**) left-hand side: depiction of the phantom samples (top) and setup with a stationary placeholder for solid sample placement (bottom). (**b**) right-hand side: schematic representation of the measurement setup with varying thicknesses of the glioblastoma phantom, denoted as alpha [1.2 mm, 2.25 mm, 3.45 mm, 4.5 mm, 8 mm] (top), and the measurement of the obscured glioblastoma phantom by PPIX-free samples with thickness beta [1.5 mm, 2.2 mm] (bottom).

The presence of PPIX within the tumor bed depends on the amount of neoplastic cells per volume, variations in the metabolic pathway of 5-ALA, and duration from 5-ALA administration leading to varying observable concentrations. For this study, PPIX was dissolved in acetone in three concentrations - high concentration with 9.1 μg/mL, medium concentration with 4.5 μg/mL, and low concentration with 1 μg/mL - and filled into 1 mL samples in Eppendorf tubes. Based on quantitative investigations of PPIX concentrations in GBM tissue, the samples should depict a meaningful range of PPIX concentrations that encompasses both vivid tumor regions (high concentration) and infiltrative zones (low concentration)^28–30^. A sub-mm height-adjustable precision slide was equipped with an Eppendorf tube exchange fixture and the provisional fluorescence detector assembly was centered under the samples (Fig. 3a). Photodiode-captured light intensity was recorded in 6 equidistant steps between 0 mm and 12.5 mm for all PPIX samples and a reference sample of pure acetone.

### Measurement setup - gelatin phantom

The effectiveness of a monitoring concept based on light interactions is essentially impacted by the propagation medium of the signal. In an inhomogeneous medium, which includes bio-tissue, absorption and scattering effects attenuate the signal ^31^. We fabricated a tissue phantom with matched glioblastoma-specific optical properties to assess the capabilities of the fluorescence detector assembly in exciting and detecting fluorescence under realistic optical conditions. Gelatin was chosen as the basic building block for the solid phantom matrix due to its versatility in adjusting scattering and absorption through additives and its negligible contribution to absorption at blue wavelengths^32^. Water with 10% gelatin volume was mixed with different amounts of titanium dioxide (TiO_2_) to adjust the scattering and India Ink to adjust the absorption. The optical properties (absorption coefficient μ_*a*_ and scattering coefficients μ_*s*_ ) at 405 nm wavelength were determined according to the method described in^33^ using a laser diode (PPM110 405-125, Power Technology, USA) at constant 50 mW and a power meter (PM100USB Power and Energy Meter, Thorlabs, Inc., New Jersey, USA). The final phantom recipe was set at 5mg/mL TiO_2_ and 3mg/mL India Ink with corresponding measured scattering coefficients of 2.9 cm$^{-1}$ and absorption coefficients of 0.8 cm$^{-1}$, which falls within the range of optical parameters of in-vivo glioblastomas according to^34^.

To manufacture the phantom, 1000 mL of water was heated to 70$^{\circ }$ C, and 130g of gelatin (10% volume with a gelatin density of 1.3g/cm$^{3}$) was dissolved in it with continuous stirring until a homogeneous solution was obtained. Following that, 5g of TiO_2_ powder and 3g of India Ink were introduced into the gelatin solution. The resulting mixture was divided into two containers, with volumes of 515 mL and 485 mL. Once the solution cooled to 30 $^{\circ }$C, 0.33 mg of PPIX dissolved in 15 mL of acetone was added to the 485 mL gelatin base to simulate PPIX-containing glioblastoma tissue with a PPIX concentration of 10 μg/mL. The remaining part served as reference tissue without PPIX. The contents of both containers were thoroughly mixed, facilitating the diffusion of settling TiO_2_ just prior to solidification.

To prepare the test samples, the mixtures were poured into round molds for curing, resulting in uniform thin sample slices with thicknesses of 1.2 mm and 2.25 mm for the glioblastoma phantom, and 1.5 mm and 2.2 mm for the reference phantom, respectively (Fig. 3b top left). Additionally, thicker phantom slices with a thickness of 8 mm were cast for both the glioblastoma and reference phantoms. The samples were then stored in the refrigerator overnight for complete curing.

In the measurement range addressed here, the comparability and reproducibility of the measurements are to a large extent conditioned by the propagation path of the excitation and emission light. For maximum consistency in this setup, a non-reflective placeholder with a round aperture was positioned over the detector assembly (Fig. 3b bottom left). During the measurement, the aperture with a diameter of 25 mm is fully obscured by the phantom sample (diameter 50 mm). This configuration guarantees a consistent traveling distance of light $\delta$, which measures 8 mm. $\delta$ results from subtracting the 2 mm height of the sensor surface from the 10 mm height of the placeholder (see Fig. 3b, top right and bottom right). Furthermore, this setup ensures a uniform exposure area, angle of incidence, and illumination power across all measurements. Two variations of the experimental execution are outlined in Fig. 3b. First, light intensity was measured in glioblastoma phantom slices, individually and then stacked, resulting in slice thicknesses of 1.2 mm, 2.25 mm, 3.45 mm, 4.5 mm, and 8 mm (denoted as $\alpha$ in Fig. 3b top right corner). The 8-mm-thick reference phantom was placed on top of the samples to simulate healthy posterior tissue. In the second set of measurements, reference slices (sample thickness denoted as $\beta$=[1.5 mm. 2.2 mm] in Fig. 3b in the bottom right corner) were placed under the 8-mm-thick glioblastoma phantom to investigate the possibility of detecting concealed cancerous tissue.

### Measurement setup - biological sample

Healthy tissue presents inherent disparities compared to the utilized optical phantoms, extending beyond optical characteristics. Firstly, the presence of autofluorescence, originating from endogenous fluorophores like collagen or flavin, has the potential to superimpose the PPIX signal. Secondly, the interplay of surface curvature, non-smooth surface structures, and the presence of blood - a known absorber of blue light - results in intricate patterns of reflection, absorption, and scattering. The detection capability of PPIX fluorescence was therefore tested on three halves of porcine brains.

**Figure 4:**
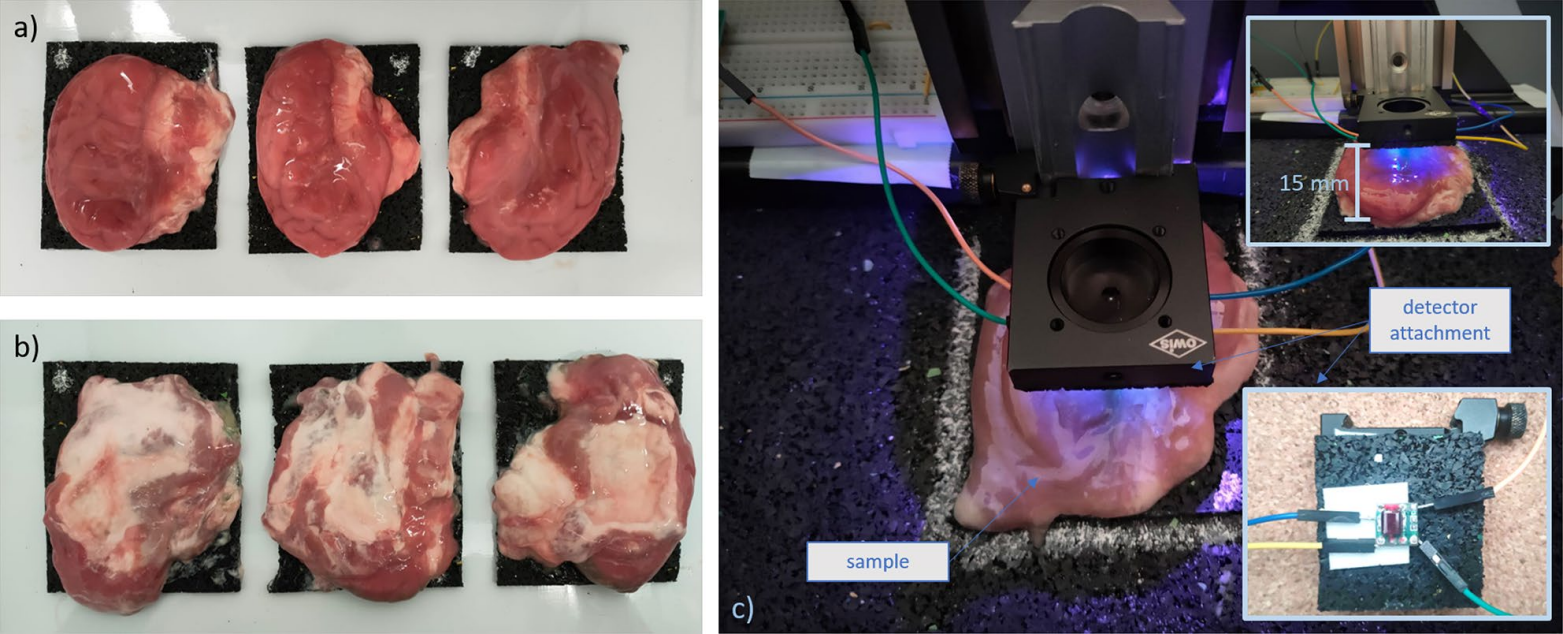
Experimental setup for fluorescence detection in biological tissue: (**a**) porcine brain tissue samples - cortex facing up; (**b**) porcine brain tissue samples - white matter facing up; (**c**) sensor and sample placement.

The brains were placed under the sensor, which was mounted at a height of 15 mm (Fig. 4c). All measurements were conducted inside a light-tight box lined with matte black rubber. Initially, signals from untreated samples were recorded. Subsequently, 0.1 mL of PPIX solution at a concentration of 10 μg/ml was injected with a syringe approximately 1 mm below the meninges at the center of the tissue, and a measurement was taken again (Fig. 4a). Afterwards, the samples were flipped to expose the white matter (Fig. 4b), and the measurements before and after the injection were repeated.

### Design deliberations

**Figure 5:**
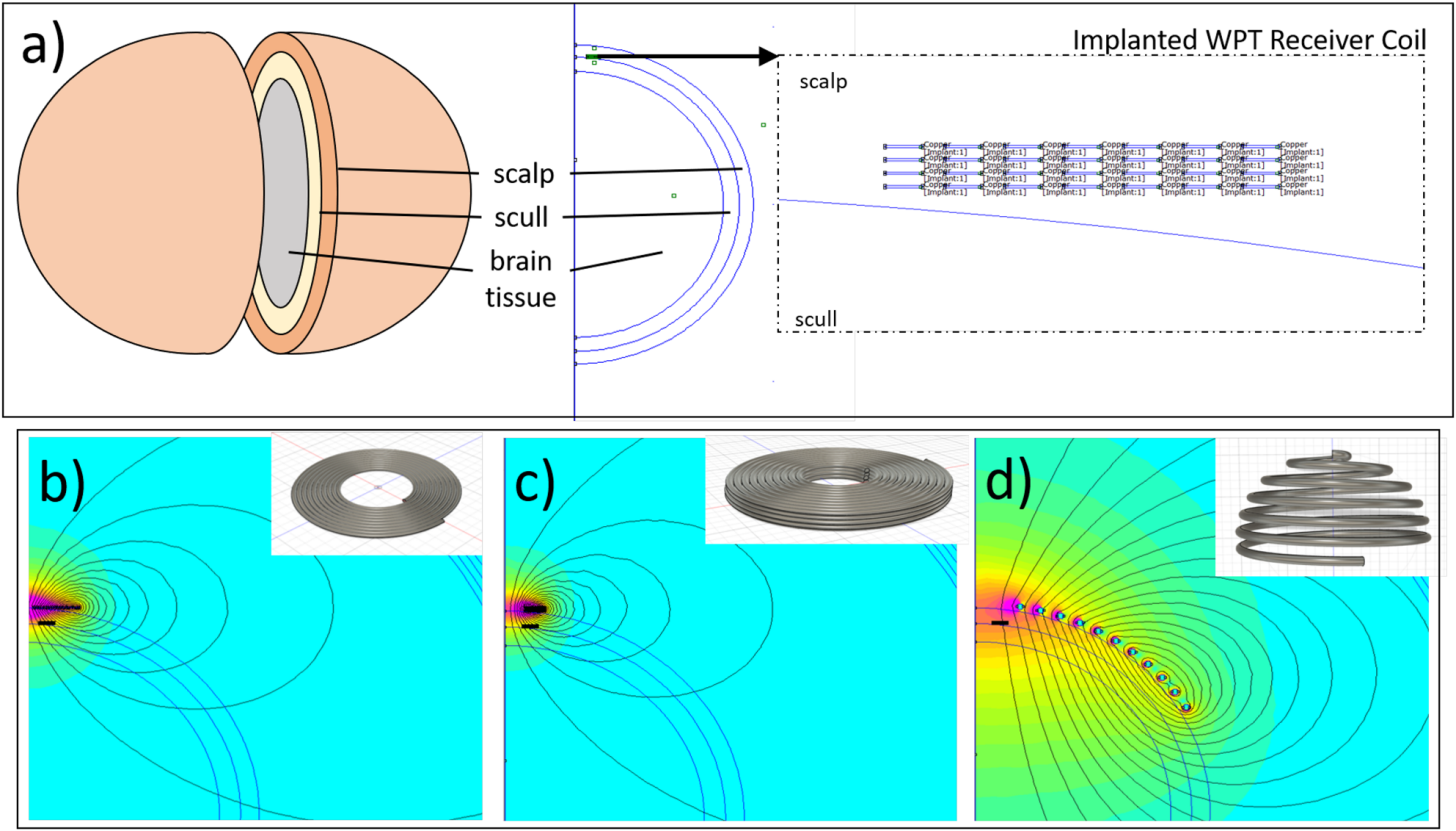
(**a**) Implementation of an axisymmetric subcutaneous wireless power transfer problem in FEMM 4.2. Representation of the coil topology of the (**b**) flat coil, (**c**) layered coil, (**d**) hemispherical coil, and the magnetic field propagation radiating from them through the head model.

The conceptual design was presented in the Introduction. To provide the necessary power for LEDs and signal conditioning during measurements, a battery will be integrated for intermediate energy storage. To enable long-term utilization of the implant, wireless transcutaneous charging of the battery is intended. The transmission of energy through tissue by the magnetic field was simulated using Finite Element Analysis (FEA) and the software tool FEMM 4.2. An abstracted human head model, consisting of a multilayered sphere was used to model the implant concept as an axisymmetric problem in two dimensions. Here, the layers represented, from the outside to inside, the tissue layers scalp, skull bone and, brain and were assigned the respective electromagnetic conduction properties of bio-tissue, 0.33 S/m, 0.0042 S/m and 0.33 S/m^35^ (Fig. 5a). A miniaturized 4-layer PCB spiral copper coil was designed and positioned in the model above the skull bone and covered by the scalp to represent the implant integrated wireless power transmission (WPT) receiver coil. Simulations were carried out with three exemplary coil topologies - a flat coil, a 4-layer spiral coil, and a hemispherical coil (shown in Fig. 5b–d) - for the extracorporeal power transceiver side at an excitation of 1 MHz. From the FEA, the frequency-dependent inductive and resistive properties as well as the coupling between the power receiver coil in the implant and transceiver coil through the bio-tissue were found.

**Figure 6:**
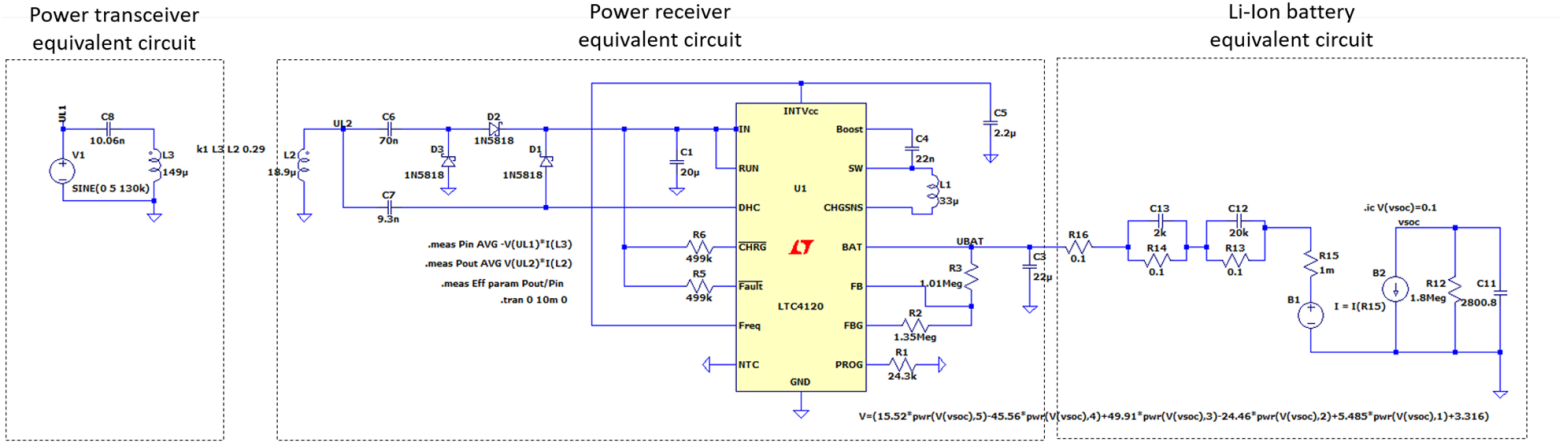
Charging circuit schematic including inductively coupled power transmission coils, charge management IC, and Li-Ion battery equivalent circuit.

The results of the field simulations were transferred to a SPICE circuit simulation as lumped parameters of a charging circuit in order to determine potential power transfer at a realistic load. The circuit is depicted in Fig. 6 and includes the coupled transceiver and receiver coils, a charging control circuit based on a battery charger IC (LTC4120, Analog Devices), and a connected Li-ion battery represented by an equivalent circuit according to^36^. The proposed circuit aims to achieve resonant inductive coupling by tuning both the primary side (comprising the extracorporeal supply circuit including the transceiver coil) and secondary side (comprising the implant integrated battery charging circuit that includes the receiver coil) to a resonant frequency of 1 MHz through the incorporation of appropriate compensation capacitances.

**Figure 7:**
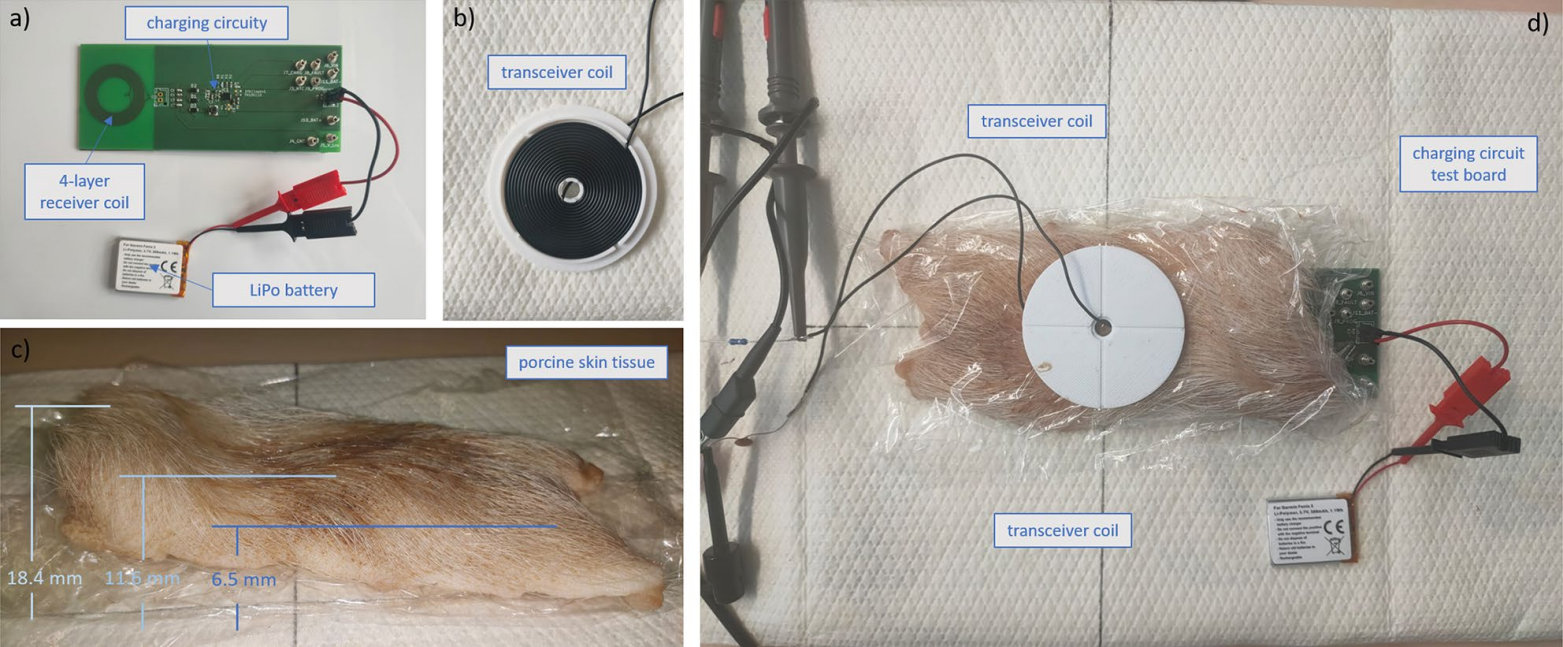
Experimental setup for transcutaneous wireless charging validation: (**a**) wireless charging evaluation circuit board; (**b**) wireless energy transceiver coil; (**c**) porcine skin (**d**) measurement setup.

The circuit was subsequently implemented on a test board (Fig. 7a) to validate the simulation results with the most promising transceiver coil geometry. The flat spiral transmission coil was wound on a custom 3D-printed holder according to the specifications in the simulation (geometric parameters are listed in Table 1, a depiction can be seen in Fig. 7b). The electrical properties of the transceiver and receiver coils were determined using an impedance analyzer (Analog Discovery 2 - 100 MS USB Oscilloscope, Digilent, Pullman, WA, USA). To simulate the scalp, a piece of porcine skin measuring approximately 6 cm × 15 cm was placed between the receiver and transceiver coil (Fig. 7c and d). The thickness of the porcine skin at the location of the coils was approximately 11.6 mm. To measure the power transfer efficiency, the transceiver coil was soldered together with a tuning capacitor and operated with a function generator (33210A Function/Arbitrary waveform Generator 10MHz, Keysight, Santa Rosa, CA, USA) at frequencies ranging from 850 kHz to 1.15 MHz and a peak voltage of 10 V. The voltage measurements and indirect current measurements using shunt resistors were also performed using the Analog Discovery 2 oscilloscope.

All functionally relevant system components were finally virtually interconnected and schematized in the ECAD software KiCad EDA (KiCad version 6.0.10, 2022) and a corresponding PCB layout was created. A three-dimensional representation of the PCB was generated and fitted with an enclosure using 3D modeling in Fusion 360$^{\circ }$ (Fusion 360 v. 2.0.13162, Autodesk, San Francisco, California, U.S.).

## Results

### Experimental investigations

**Figure 8:**
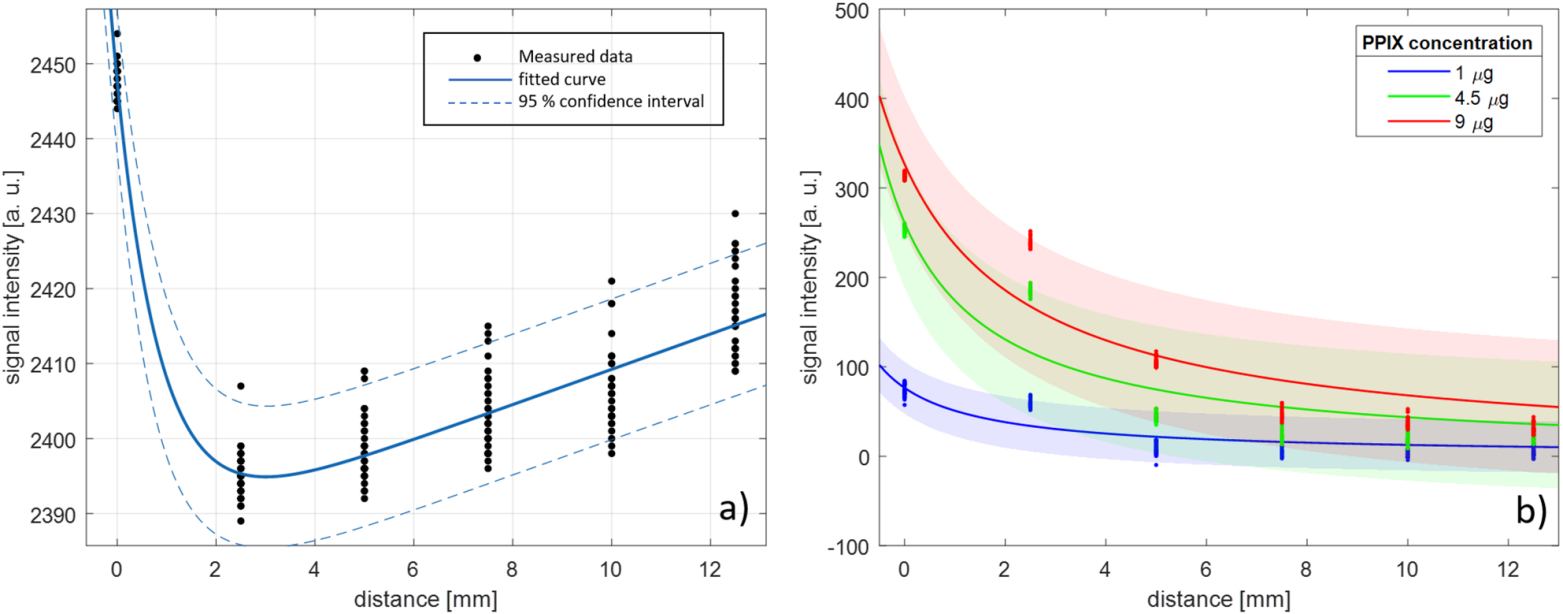
(**a**) Light signal intensity recorded at different distances between the PPIX-free reference sample and photodiode. (**b**) Cleaned intensity signals of the liquid PPIX-samples vs. photodiode distance.

Figure 8a shows the measurement data obtained from the reference measurement with the PPIX-free liquid acetone sample. The graph plots the light intensity (in arbitrary units, represented as a bit representation of the system- and gain-dependent voltage at the ADC) received by the photodiode against the distance of the sample from the photodiode surface. The error-ridden measurement curve closely follows a second-degree exponential model ( β1 = −1.13, CI1: -1.43 – 0.82, β2 = 0.001, CI2: 0.0008 – 0.00011, R2 = 0.93) which is depicted as a blue curve with corresponding dashed 95 % confidence boundaries. The reference measurement curve is caused exclusively by PPIX-fluorescence-independent phenomena in the measurement set-up, such as non-filtered light components of the blue excitation light, reflections from Eppendorf tubes and the acetone contained therein, or shadows cast by the sample. Determining the reference curve allowed to eliminate the background noise baseline from the measurements with PPIX samples leaving the fluorescence signal only for further analysis. In the implantation scenario, it implies that measurements are always referenced to a baseline measurement, which is determined immediately after the resection and implantation of the detector. The noise-corrected measurement points recorded from the PPIX-containing samples can be seen in Fig. 8b. The fluorescence intensity follows a reciprocal course as a function of the distance and was fitted by rational modeling with:

- p$_{1}$ =152.3 (CI 127.1–177.6); q$_{1}$ = 1.992 (CI 1.615 –2.37); R$^{2}$ = 0.76 for 1 μg/mL PPIX;
- p$_{1}$ =523.2 (CI 460–586.4); q$_{1}$ = 2.006 (CI 1.729 –2.283); R$^{2}$ = 0.85 for 4.5 μg/mL PPIX;
- p$_{1}$ =860.4 (CI 779.8 – 940.9); q$_{1}$ =2.636 (CI 2.344 –2.927); R$^{2}$ = 0.76 for 9 μg/mL PPIX

At small distances between the sample and the sensor, significant signals were recorded that allowed for the identification of the PPIX concentration present in the sample. The signal intensity drops rapidly with increasing distance from the sample. At a distance of 2 mm, the signals have already decreased by 43%, 50%, and 50% for concentrations of 9 μg, 4.5 μg, and 1 μg, respectively. The significance of these values can only provide a rough estimate and should be interpreted with extreme caution. Firstly, in future development steps, measures can be taken to increase the sensitivity of the detector and reduce measurement errors. These will be explained in more detail in the Discussion. Secondly, this measurement does not represent a realistic scenario in any way. In in-vivo resection cavity measurements, numerous other influences affect the signal, such as autofluorescence in healthy tissue, natural changes in the resection cavity volume, and optical effects such as reflection, scattering, and absorption of light signals.

However, the overall measurement result demonstrates how the proposed LED-photodiode-based detection module is capable to respond to even minor PPIX presence at reasonable sample distances and capturing both concentration and distance-dependent signal variations within the required sensitivity range.

**Figure 9:**
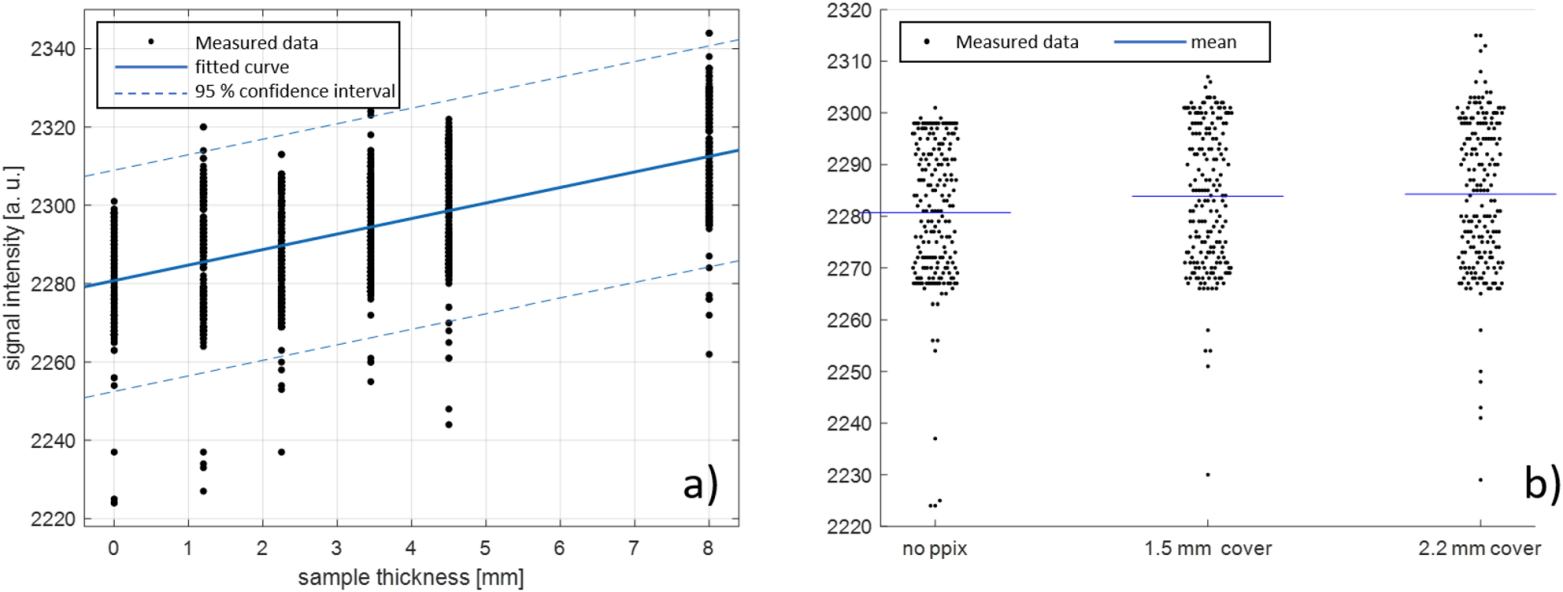
(**a**) Light signal intensity recorded from gelatin phantom samples with different thicknesses. (**b**) Intensity measured at PPIX-free reference phantom, and PPIX-containing phantom covered with PPIX-free phantom.

Figure 9a shows the signal intensities from the PPIX-containing gelatin phantoms as a function of their thickness, with the PPIX-free 8 mm thick gelatin phantom included in the graph as an additional measurement point at the thickness of 0 mm. In the measured layer thickness range, a linear relationship can be observed between the emitted PPIX fluorescence and the thickness of the sample, illustrated by the blue regression line including its 95 % confidence interval (β = 3.97, CI 3.65 – 4.29, R2 = 0.34). Figure 9b shows the measurements from the second experimental setup incorporating gelatine phantoms. The measured light intensities of the 8 mm thick PPIX-containing sample covered by a 1.5 mm thick PPIX-free gelatine phantom slice (middle cluster of points) and a 2.2 mm thick PPIX-free gelatine phantom slice (right cluster of points) are shown. For the convenience of interpretation, the PPIX-free 8mm phantom was included in the graph as a reference (cluster on the left) and the mean values of all measurements were inserted as blue lines at 2280.7, 2283.9, and 2284.3 from left to right. The slightly higher expected values (mean values) measured at the covered PPIX-containing phantom samples suggest that fluorescence detection through thin PPIX-free tissue layers might be possible using a photodiode sensor. However, due to the high scatter of the measurement values, no clear statement can be made with the underlying data. Furthermore, a higher expected value was determined with the thicker coverage, which contradicts signal attenuation mechanics in tissue.

A direct comparison of the measurement values obtained with biological samples, both among themselves and to the measurements from previous experiments, is not possible due to the unequal experimental conditions resulting from the natural heterogeneity of biotissue. One crucial factor is the variable distance between the sensor and the sample surface, which is determined by the sample thickness and the different surface curvatures. These factors strongly influence the excitation light reflections, which are reflected in the high fluctuations of the reference values before injection. In comparison to the gelatin experiments, the reference signal is significantly higher, which can be attributed to the larger reflective surface and the shorter distance to the sensor ($\tilde{2}$ mm–5 mm). Furthermore, the distribution pattern of the injected PPIX solution in the tissue of individual samples is unknown. A uniform, concentrated accumulation of the fluid is likely to result in a stronger signal than a diffuse spread into distant regions from the injection site.

**Figure 10:**
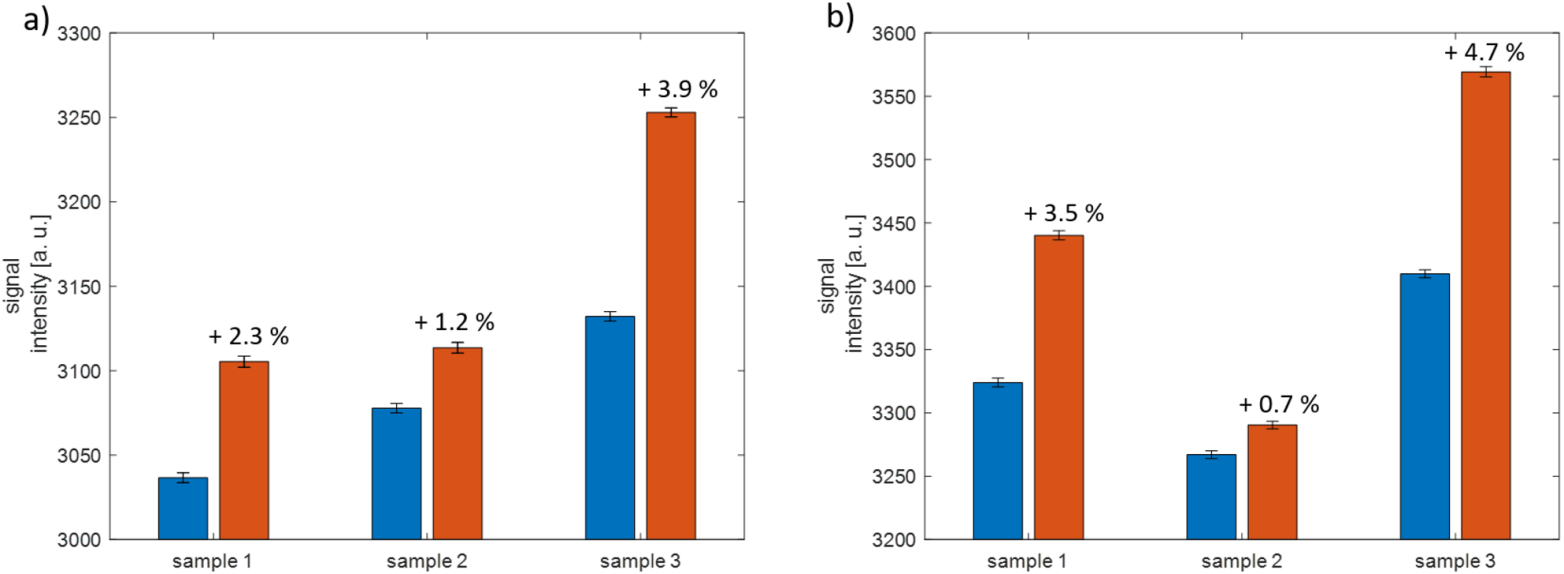
(**a**) Light signal intensity recorded from porcine brain grey matter before (blue) and after (red) injection with PPIX. (**b**) Light signal intensity recorded from porcine brain white matter before (blue) and after (red) injection with PPIX.

Under these limitations, it can be deduced from the measured signals that a distinct signal can be detected in both gray and white matter, with a clear signal separation to the reference measurement (Fig. 10a and b). The differences in the optical properties of brain tissue are reflected in the reference signals, with significantly higher values in white matter (ranging from an average of 3324 to 3410) compared to gray matter (ranging from an average of 3037 to 3132). However, no relevant differences can be observed in the useful signal, which is measured as the difference between the post-injection signal and the pre-injection signal. These differences amount to 69, 36, and 121 for gray matter, and 117, 23, and 169 for white matter.

By comparing the relative measurement values from the biological tissue with the measurements of PPIX in acetone, the scale of effects such as tissue optical properties and background fluorescence on measurability can be deduced. While signals between 186 and 113 are expected in the measurement of PPIX in a clear solution with a sample-sensor distance between 2 and 5 mm, three of the signals from biological tissue are well below this range, and two are at the lower end.

### Wireless power transmission modeling

**Figure 11:**
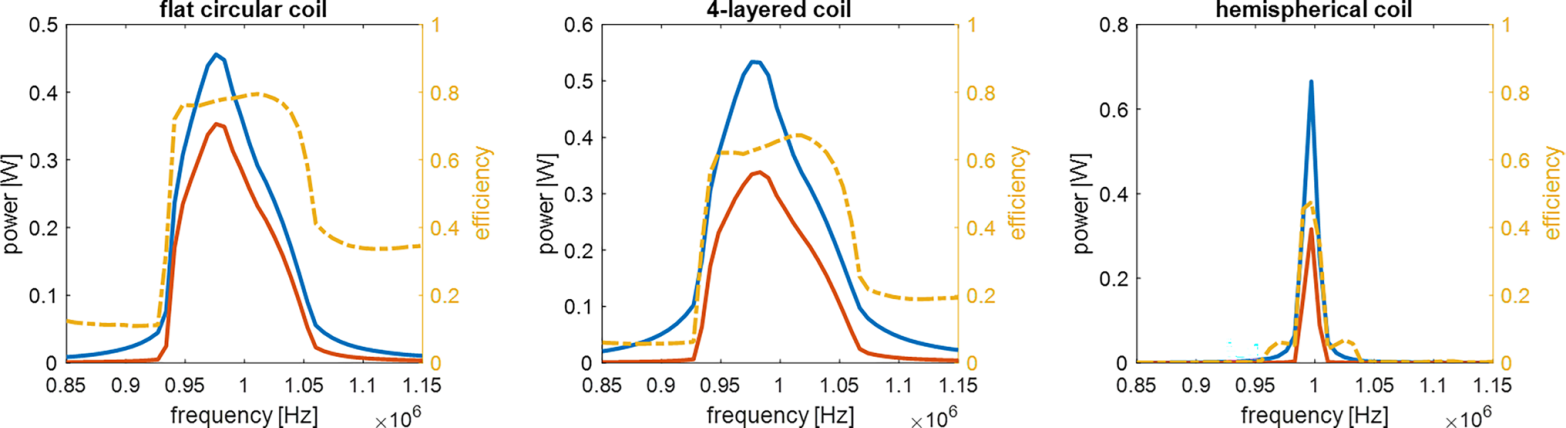
Simulated power transmission characteristics of the three investigated transceiver coil topologies with the input power (blue), transferred power (red), and transfer efficiency (orange) vs. input frequency.

Table 1 summarizes the geometrical parameters and simulation results for the implanted receiver coil and the different extracorporeal transceiver coils. The primary objective of the simulations was to demonstrate the feasibility of wireless implant power supply on the basis of three exemplary coil topologies. A direct comparison of the coils with each other is not meaningful in this form. The geometric parameters were determined at random and thus, completely different results can arise with an optimization-oriented design. In addition, characteristics such as omnidirectionality were not taken into account at all. Given these constraints, it can be concluded that the 4-layer spiral design of the transceiver coil at 143 μH and 31 $\Omega$ leads to a comparable coupling factor to the larger diameter flat coil design, which will presumably lead to lower heat losses due to the reduced resistance and higher coil quality factor. The hemispherical topology, which is comparatively insensitive to alignment, has a very high coil quality factor but achieves a significantly poorer coupling to the implant. This means that a large part of the magnetic field does not penetrate the receiver coil and is potentially absorbed by the bio-tissue. Figure 11 shows the input power (blue curve), the power transferred to the receiver coil (red curve), and the transfer efficiency (orange curve) associated with the three simulated transceiver coil topologies around the resonant frequency of the circuit. In all three configurations, a significant increase in the transmitted power is seen at the tuned resonance frequency. With the hardware configuration used in the simulation, high efficiencies of 0.8 and 0.7 respectively can be achieved with the flat and multilayer coil topology while the hemispherical coil reaches a maximum of 0.47.

**Table 1:** Geometrical parameters of FEA-simulated implanted receiver and extracorporeal transceiver coils and resulting coil properties at 1 MHz.

	Receiver	Transceiver		
		Design 1	Design 2	Design 3
Topology	4-layer spiral	flat circular coil	4-layer spiral	Hemisphere
Windings	28 (7 per layer)	18	19 per layer	11
Diameter	25 mm	44 mm	30 mm	160 mm
Conductor cross-section	35 μm $\cdot$ 500 μm	$\pi \cdot$ (150 μm)$^{2}$	$\pi \cdot$ (150 μm)$^{2}$	$\pi \cdot$ (1.75 mm)$^{2}$
Inductivity at 1 MHz	18.9 μH	7.5 μH	143 μH	9.57 μH
Resistance at 1 MHz	1.85 $\Omega$	0.8 $\Omega$	31 $\Omega$	0.33 $\Omega$
Coil quality factor	64	59	29	182
Coupling Coefficient	–	0.3	0.29	0.09

**Figure 12:**
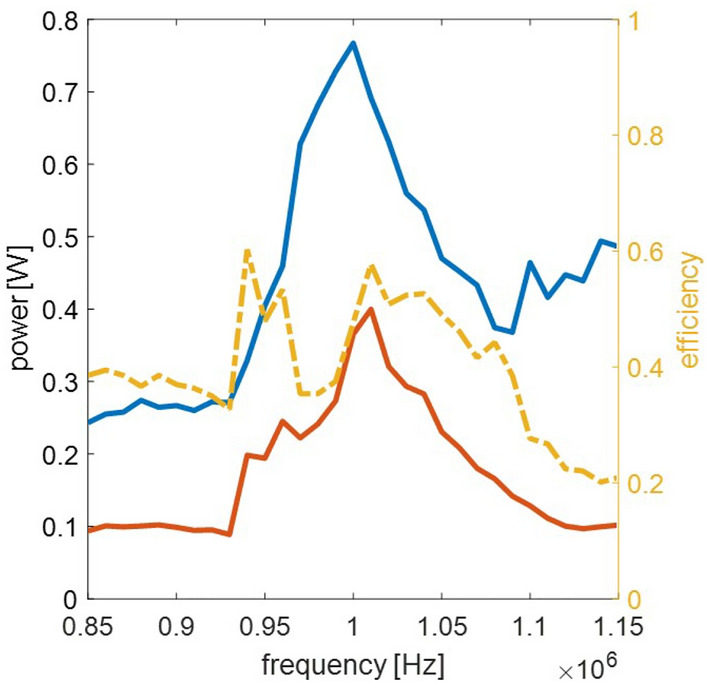
Measured power transmission characteristics including input power (blue), transferred power (red), and transfer efficiency (orange) vs. input frequency.

Due to its overall balanced electrical properties and promising simulated transfer efficiency, the flat spiral coil was replicated for a charging circuit test. The results are shown in Table 2 and Fig. 12. The measured inductance and resistance of the receiver coil are higher than the simulated values (8.7 % and 4.9 % respectively), resulting in an overall slightly higher coil quality of 66. In the transceiver coil, a 15.3 % lower inductance and simultaneously a twice as high resistance value were measured, leading to a significantly lower coil quality factor of 34. The coupling factor, which depends primarily on the geometric orientation of the coils and the composition of the magnetic field propagation medium, is 15 % lower than the simulation value, with a value of 26. This can be attributed to the greater thickness of the tissue sample, around 11 mm, compared to the 7 mm scalp thickness used in the simulation.

The power measurement results show a less pronounced power peak at the resonance frequency compared to the simulation results. This behavior is closely related to the coil quality, which is lower due to the higher resistances observed during testing. Additionally, a shunt resistor of 1 Ohm was used in the measurements to capture the current, which further reduces the coil quality and the associated transfer efficiency.

Overall, wireless charging through biotissue was successfully achieved with the circuit, achieving an efficiency of up to 0.6 around the resonance frequency whereby the higher tissue thickness compared to the simulations should be taken into account.

**Table 2:** Measured coil electrical properties.

	Receiver	Transceiver
Inductivity at 1 MHz	20.55 μH	6.35 μH
Resistance at 1 MHz	1.94 $\Omega$	1.17 $\Omega$
Coil quality factor	66	34
Coupling Coefficient	–	0.26

### Overall concept

**Figure 13:**
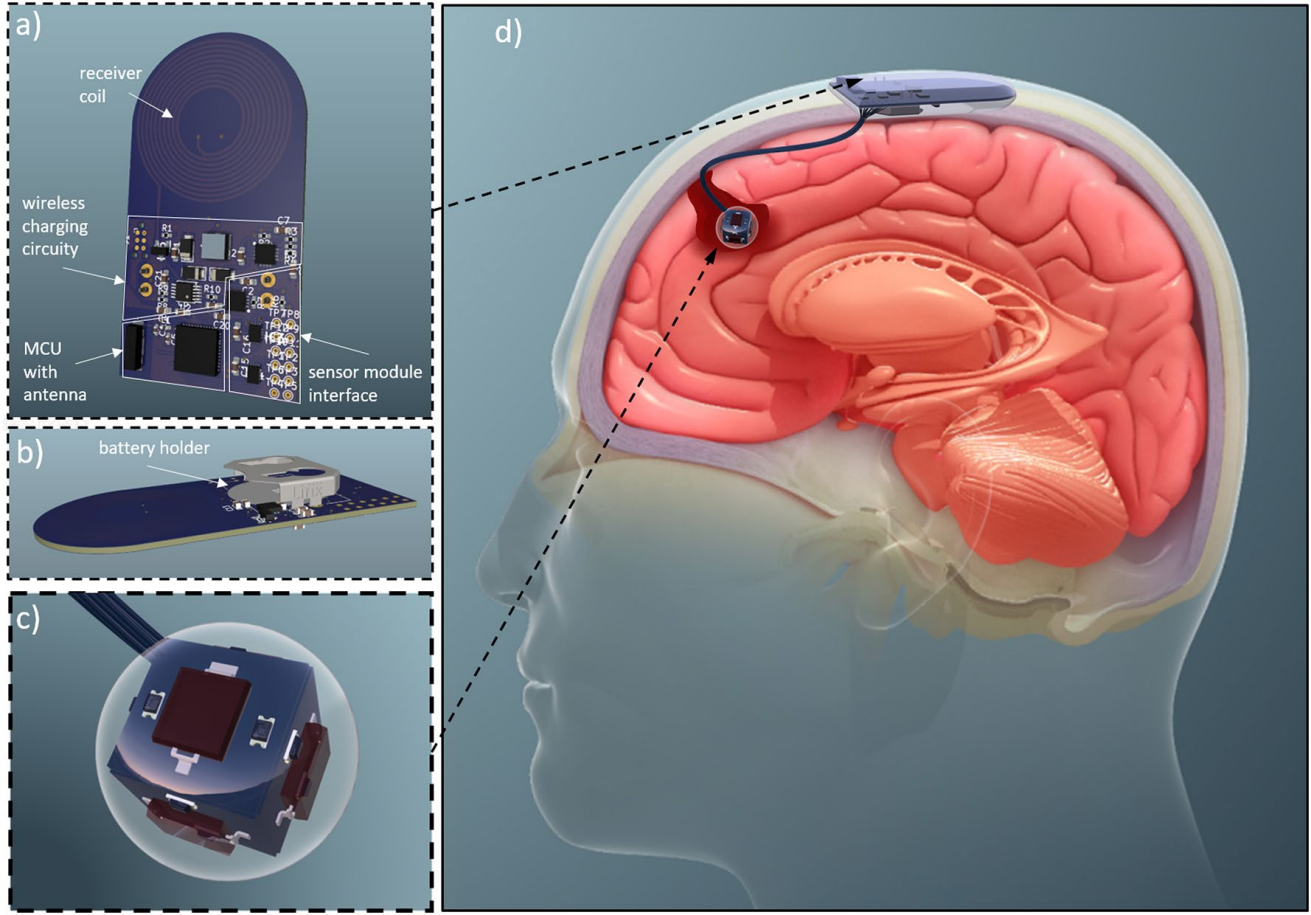
Visualization of the envisioned implantable cancer tissue detection system based on previously tested or simulated electronics. The image on the right is adapted from “A depiction of various types of cerebral Hematoma(L to R) - Epidural Hematoma, Subdural Hematoma, and Intracranial Hematoma.” by www.scientificanimations.com licensed under CC BY-SA 4.0. The image has been cropped and items have been added.

Figure 13 illustrates the overall concept of the implant for monitoring tumor recurrence. The visualization is based on the electronic circuits utilized in the experimental setup and the electronic circuits employed in the energy analyses. To complete the system functionality, a microcontroller (MC) chip and two multiplexers were added to the computing unit hardware (Fig. 13a). The selected MC chip (ESP32-PICO-V3-02, Espressif) possesses Bluetooth Low Energy (BLE) communication capabilities, enabling it to transmit measurement data to an extracorporeal charging and readout unit in addition to controlling the measurement process. BLE is particularly suitable for battery-operated systems due to its low power consumption. It is utilized and approved for transcutaneous data transmission in multiple commercial implantable systems including cardiac monitors^37,38^, pacemakers^39^, and glucose monitors^40^. The multiplexers serve as an interface between the computing unit and the detector module, allowing for separate control of individual LEDs and sensor elements.

All circuit components were interconnected on a PCB layout, which was subsequently transformed into a 3D representation of the computing unit, as illustrated in Fig. 13a. The spiral power receiver coil is evident in the upper part of the PCB and extends over the four layers of the PCB as specified in the simulations. The PCB dimensions, and therefore the approximate size of the subcutaneous computing module, are 56.8 mm × 30.04 mm. In this implementation, a standard-size LIR2032 Li-Ion coin cell battery is intended to be placed on the backside of the PCB. With the battery holder and electronic components on both sides of the board, the maximum depth dimension is 7.59 mm. The sensor module consists of six discrete circuit boards, each with a photodiode and two LEDs, which are combined into a cube to sequentially scan the resection cavity from 360$^{\circ }$ (Fig. 13b). The edge length of the cube is 8.3 mm and the sensor module is placed in a clear spherical enclosure with a diameter of 15 mm.

The implantation procedure is illustrated in Fig. 13c. Following tumor resection and achieving hemostasis during the resection procedure, the detector module is positioned within the resulting resection cavity and securely affixed using fibrin sealant. A slender and flexible cable connects the detector module to the computing module, which is threaded and sealed through the dura mater during dural closure. A small aperture at the periphery of the excised bone flap facilitates the connection through the skull. Prior to the closure of the scalp layers, the computing module is situated and firmly secured onto an intact region of the skull.

To conduct a measurement, oral administration of 5-ALA needs to be performed four to eight hours prior to the procedure^30^.To obtain meaningful measurements, it is important to consistently maintain the same duration between administration and measurement. The electronics are engineered to maintain a passive state when not in use, with the power source decoupled from the measurement circuitry. This minimizes the risk of electrical hazards associated with active implants and safeguards against battery discharge caused by leakage currents. Upon application of an external magnetic field by a complementary extracorporeal unit for charging and readout purposes, the charging process, regulated by the battery charger IC, commences automatically. Once the charging process is completed, the charger IC sends a signal, establishing a connection between the battery and the measurement circuity. By means of the multiplexers, the microcontroller independently controls the sensor elements of the detector cube, permitting sequential measurement of the cavity environment with minimal instantaneous current. In the final phase, the microcontroller transmits the acquired measurement data to the extracorporeal unit. Following confirmation of successful data transmission, the microcontroller activates a switch to decouple the power supply of the measurement circuit, reinstating the system to a passive state. Subsequently, the data can be transmitted to the treating physician for evaluation. By analyzing the temporal evolution of the signals the physician can infer tissue changes within the brain region and that can aid decision making on the further course of the therapy.

## Discussion of the results and related follow-up activities

In both the liquid and solid test samples, fluorescence was induced by the radiant power of small-sized SMD diodes at a level that produced a measurable signal at the photodiode. The acquired data revealed a reciprocal relationship between signal intensity and sample spacing as well as a linear relationship between signal intensity and sample thickness and a strong correlation with PPIX concentration. In biological brain tissue as well, distinct signals could be detected, with the increased reflection of the excitation light in white matter leading to significantly higher background noise levels.

Based on these outcomes, the feasibility of the proposed sensing approach for the detection and monitoring of recurrent glioblastoma tissue could be demonstrated. However, to achieve an adequate resolution required to determine minor histological changes, the measurement uncertainty needs to be reduced significantly and the measurement sensitivity needs to be increased. A significant improvement in this regard will be achieved through the subsequent fabrication of the PCB-based prototype shown in Fig. 13a. In contrast to the measurement electronics used in the experimental setup, enormous sources of noise and measurement errors such as long lines with susceptible metal clip connections, non-compensated parasitics in the plug-in board, or a non-stabilized supply voltage are eliminated. Further, a simple red gel filter has been employed in the presented setup, which passed a substantial portion of the blue excitation light, several times larger than the useful fluorescence signal. In the next development step, a dedicated optical filter, such as a thin-film interference filter with a short-wave rejection of close to 100 %, will be installed. The filtered-out fluorescence signal can thus be carried at an increased gain without driving the amplifier into saturation, thereby significantly increasing sensor sensitivity.

Moreover, the investigations into the detection capability presented in this study through the proposed concept have significant limitations. The experimental tests conducted herein offer a highly constrained representation of in-vivo measurements within a resection cavity. In addition to structural and volume changes post-procedure, the resection cavity becomes filled with cerebrospinal fluid, which acts as a fluorescence signal propagation medium between the sensor and the tissue surface. This introduces substantial signal attenuation along the light signal path. Furthermore, necrosis and scar formation are common side effects of adjuvant radiotherapy, which may alter the optical properties of the tissue. To specify the sensitivity and accuracy of the detector, future development stages must undergo extensive testing in a series of comprehensive multi-scenario experiments incorporating possible variations of biotissue.

Particularly from measurements with biological tissue, it has become evident that the measurement value alone cannot provide a direct inference regarding the amount of cancerous tissue. Interpretation of the measurements can only be done through comparative measurements to an initial baseline measurement and temporal changes. An important criterion for this is a stable and immobile position of the detector within the resection cavity to ensure that the same measurement field is captured by the same sensor element. In the further development process, solutions for permanent and reliable stabilization need to be investigated. Potentially, miniaturized tacks or anchors could be implemented as extensions of the encapsulation. In order to provide meaningful information about the detection capability and interpretation of measurement values, the natural reduction of the resection cavity volume and false-positive signals, which can occur, for example, in edemas, need to be simulated and evaluated in the future.

Appropriate implementation of the system, allowing for precision measurements exceeding the measurement accuracy reported here, and testing under various scenarios will be the focus of follow-up work.

It has been shown by the field simulations that inductive coupling occurs through the tissue layers of the head and energy transfer is achievable with different designs of the extracorporeal transfer unit. Even with the comparably low coupling factor of 0.09, energy transfer can be successfully established with methods like magnetic field repeater circuits^41^. The SPICE simulations firstly show that the energy transfer can be realized at a reasonable efficiency and a successful charging process through biotissue was performed in the test. The circuit functionality has been verified in a test with power transfer through porcine skin tissue. During the measurement process, two LEDs (2x20 mA^42^), the amplifier circuit (15.5 mA^43^), the ADC (0.24 mA^44^), and the microcontroller (31 mA^45^) consume power. The multiplexers only draw a negligible leakage current. Therefore, the measurement process requires approximately 87 mA, and the system could be operated for over 30 minutes using a battery capacity of 45mAh^46^. The acquisition of a measurement point takes place in the millisecond range. For example, in our experiments, a measurement point was recorded every 600ms. In one measurement cycle that is always initiated with a complete charging of the battery, it would be possible to capture up to 3000 measurement values at this sampling rate.

With an area of 56.8 mm × 30.04 mm, the computing module is comparable to the dimensions of commercial Cochlear implants (56.2 × 28.5 mm^2^^47^, 45.7 × 25.4 mm^2^^48^). Due to the battery on the PCB backside, the thickness of 7.59 mm exceeds the thickness of 4.5 mm of comparable implants by almost double. Potential solutions to be considered further include moving the battery to the front side and thus accepting an increase in surface area or using alternative energy storage modules with smaller dimensions. For example, LiPo batteries, which are available in a wide range of geometric designs and are produced by manufacturers specifically for medical implants, or supercapacitors, unlike lithium-based batteries, can also deliver high currents in very small dimensions. In the redesign of the computing module, alternatives for data transmission and signal processing will also be implemented and tested, such as the reduction of the components and AM data up-modulation to the energy transmission channel. By eliminating redundant logic blocks that are present in off-the-shelf Microcontrollers and ICs and reducing circuit complexity, power requirements can be further mitigated so that smaller energy storage units can be considered.

With a diameter of 15 mm for the detector module, only patients with a correspondingly large resection cavity are suitable for implantation. The weight of the presented detector module can be estimated at 2.72 g. The calculation includes 6 × 43 mg per photodiode, 12 × 20 mg per LED, 6 × 130 mg per side of the PCB cube (calculated using a PCB manufacturer online tool^49^ with an edge length of 8.3 mm and a thickness of 0.8 mm), and 1.44 g for the encapsulation (calculated for a Polydimethylsiloxane (PDMS) encapsulation). PDMS is a commonly utilized encapsulation material for chronic medical implants, known for its biocompatibility, biostability, excellent electrical and thermal insulation^50,51^. Moreover, PDMS exhibits favorable optical properties, surpassing glass in terms of light transmittance^52^. It has been demonstrated that a mismatch in the mechanical properties of implants, including weight, shape, and flexibility, can lead to tissue damage and cellular reactions^50^. While the flexibility of PDMS can be adjusted within a wide range, brain tissue of the detector volume, with a density of 1040 kg/m$^{3}$, weighs approximately only 1.8 g. To ensure structural biocompatibility and minimize this risk by mechanical mismatch, further studies will explore solutions for reducing the detector module weight by 34%. The mechanical interactions between implanted hardware, the bio-tissue, and the cerebrospinal fluid concerning cavity pressure, frictional stress, and strain forces have to be carefully investigated and corresponding design solutions based on biocompatible materials need to be explored.

Brain tissue is one of the most sensitive tissues to heating. With PDMS encapsulation, excellent heat insulation is ensured, allowing the assumption that the temperature rise caused by the electronics will be uniformly distributed on the implant surface^53^. The surface heat source density, predominantly resulting from two simultaneously operating LEDs, can be estimated at approximately 19 mW/cm$^{2}$ (at a diode current of 20 mA, forward voltage of 3.3 V, and a detector module surface area of 7.07 cm$^{2}$). Temperature increases in tissue caused by implanted electronics are subject to complex relationships involving factors such as duration of operation, heat loss due to perfusion, metabolic heat generation, and others. There are no defined threshold limits for maximum power density in brain tissue. However, for comparative purposes, a reference value can be drawn from a study on heating in muscle and lung tissue. In this study, implants operated continuously at 40 mW/cm$^{2}$ were examined, and no harmful effects were observed over a period of 7 weeks.

For intraoperative imaging using 5-ALA, recommended illumination light exposure levels range from 40–80 mW/cm$^{2}$^54^. In this context, the two LEDs utilized in our setup, with a maximum irradiation power of 10 mW^42^, are significantly below the recommended levels. Therefore, the risk of tissue damage can be assumed highly unlikely, particularly considering the short exposure duration involved.

Furthermore, compatibility with MRI, as the most important modality for brain imaging, is a major aspect of the further system design. MRI examinations are conditionally possible with comparable neuro implants such as the Cochlear implant, which also features a flat coil structure or the DBS Implant which introduces elongated electrodes and extension wires into the brain. In this context, most adverse effects are associated with the integrated magnet for the Cochlear implant and with the electrode-tissue interface for Deep Brain Stimulation Implants^55^, neither of which are foreseen in our concept. However, irrespective of the patient safety, the implants may cause artifacts in the resulting MR images. The concept of implantable monitoring, as it can only detect cancer in its immediate vicinity, will not obviate the need for MRI examinations that can also discover distant recurrence. On the design side, it is, therefore, crucial to ensure that potential sources of interference such as metallic parts are minimized or that new MRI-compatible materials^56^ are incorporated. Moreover, it is essential to evaluate various established metal artifact reduction techniques in subsequent development phases and formulate strategies for obtaining MRI scans devoid of artifacts.

Numerous aspects of the presented concept need to be further specified and elucidated in future research endeavors. Particularly regarding compatibility and biosafety, comprehensive and extensive investigations are required in subsequent work to ensure a thorough evaluation.

## Conclusion

Current monitoring approaches for glioblastoma resection rely on imaging techniques that allow data acquisition only at limited intervals and do not capture dynamic changes. In this study, we have introduced a radically novel surveillance approach for glioblastoma resection patients, utilizing an implantable system that enables routine remote monitoring. While 5-ALA-based fluorescence has been used for intraoperative delineation of cancerous tissue, our experimental measurements have demonstrated its potential for signaling the presence of recurrent cancerous tissue to a miniaturized intracorporal detector. We have presented a conceptual design of a spatially distributed dual-module system, which is of reasonable size and feasible for implantation, similar to existing approved implants. The realization of an implantable active monitoring device is immensely challenging on practicability and patient safety and multiple essential concerns need to be addressed and design solutions found in follow-up efforts. Nevertheless, potential improvements over conventional monitoring may lead to improved patient prognosis. Through continuous monitoring, this approach might allow earlier detection of cancerous tissue and timely resection, if necessary. The recorded data could be used to assess and adjust the course and efficacy of adjuvant therapies in a comprehensive and unambiguous manner.

## Data Availability

The datasets generated during during the current study are available from the corresponding author for reasonable request.
